# Determinants of Attitude toward the Public Health Spending and Its Relationship with Voting Behavior in the 2012 South Korean Presidential Election

**DOI:** 10.1371/journal.pone.0163763

**Published:** 2016-10-06

**Authors:** Sang Jun Eun, Jin Yong Lee, Hye-Min Jung, Jin-Seok Lee

**Affiliations:** 1 Department of Preventive Medicine, Chungnam National University School of Medicine, Daejeon, Republic of Korea; 2 Public Health Medical Service, Seoul National University Boramae Medical Center, Seoul, Republic of Korea; 3 Regional Rheumatoid and Degenerative Arthritis Center, Chungnam National University Hospital, Daejeon, Republic of Korea; 4 Department of Health Policy and Management, Seoul National University College of Medicine, Seoul, Republic of Korea; 5 Institute of Health Policy and Management, Medical Research Center, Seoul National University, Seoul, Republic of Korea; SOAS, University of London, UNITED KINGDOM

## Abstract

This study aimed to identify the factors influencing South Korean voters’ attitudes towards increasing public expenditure on health and to identify whether the issue of healthcare expenditure influenced candidate choice in the 2012 Korean presidential election. The study used the data from a survey conducted by the Institute of Korean Politics at Seoul National University immediately following the 2012 presidential election. The survey was completed by a nationwide sample of 1,200 people aged 19 or over using a face-to-face interview method and proportional quota sampling based on sex, age, and region. About 44.3% of respondents had a positive attitude toward increasing public health expenditure. There was no significant difference by the candidate they supported (conservative Park Geun-hye or liberal Moon Jae-in). In particular, even 44.9% of conservative supporters agreed with more spending. Politically neutral respondents (OR = 1.76, 90% CI 1.22–2.54) and strong conservative party supporters (OR = 1.53, 90% CI 1.05–2.25) were more likely to support public health expenditure increase compared to strong liberal party supporters. Also, respondents who believed that the economic gap in the country was widening were 1.91 times more likely to support an increase in public health expenditures. However, the issue of health expenditure had no influence on voters’ choice of presidential candidates, and in particular no negative effect of choice of the ruling (conservative) party’s candidate. Our results should be interpreted with care; one possible reason for this lack of effect might be that constituents voted along partisan lines regardless of their attitude to the welfare issue; another possible explanation might be the success of the “left click strategy” of the conservative party. That is, the conservatives did not reject economic democratization or social welfare expansion. Further research should be done to explain why attitudes to health spending did not directly affect choice of candidate.

## Introduction

“Economic democratization” and “social welfare expansion” were two major debates during the 2012 presidential election campaign in South Korea [1–3]. Korean people recognized that economic and social disparities had increased during the Lee Myung-bak administration, which had begun in 2008. These attitudes were almost certainly exacerbated by the uneven recovery from the 2008 global economic crisis. However, Lee’s “business-friendly” policy was also damaging in this regard, since it focused on supporting big businesses such as the family-controlled conglomerates (*chaebol*), while small and medium-sized companies were sacrificed and social welfare programs neglected or ignored. Likely as a result, demand for expanded social welfare spending increased. Against this background, “economic democratization” and “social welfare expansion” became the issues at the forefront of the presidential campaign [1–3].

The most important among the issues related to social welfare expansion was how to set political priorities in order to overcome the structural weak points of the National Health Insurance (NHI) scheme. National Health Insurance, a mandatory social insurance program, was launched in 1977 and had extended its benefits to the entire population by 1989 [4, 5], a remarkable achievement in only 12 years. However, many items were categorized as non-covered under the program, consequently raising the out-of-pocket burden on patients [4, 5]. According to the OECD figures, the share of total healthcare expenditure in Korea covered by individual out-of-pocket expenses was 35.9%, the second-highest among all OECD countries [6], while medical expenses as a share of total household consumption expenditure was third-highest [7]. At the same time, the proportion of public spending devoted to health (53.9% in 2007) was considerably lower than the OECD average of 73%, and NHI coverage still achieved a rate of 63.0% in 2011 [5]. In sum, the Korean government had made a decision that rapid NHI expansion for all Korean citizens in short period was its most important political priority, ahead of providing full coverage package [8]. As a result of this decision, the NHI has been criticized for purporting to be a “universal health insurance system” while offering only limited coverage and unequal access [9]. Korean patients are receiving incomplete universal health insurance coverage and still have relatively high exposure to risk and financial burden [5, 8, 9].

In the 2012 election, the incumbent conservative ruling party’s candidate was Park Geun-hye and the liberal opposition party’s candidate was Moon Jae-in. During the election campaign, these two leading candidates debated how to improve the NHI system. The conservatives insisted that expanding coverage of severe and rare diseases should be the top priority, while the liberals asserted that reducing the burden of out-of-pocket payment should rather be the focus. These respective positions on the NHI reflected different sets of broader values: the “customized welfare” upheld by the conservatives versus the “universal welfare” of the liberals [1]:

*The encounter will focus on welfare*. *So far*, *Park and Moon have both called for free day-care programs for those aged five or under*, *cuts in college tuition by 50 percent*, *and free high school–level education*. *However*, *Moon has suggested more extensive health insurance subsidies than Park [in that] he has pledged to restrict the maximum amount a patient will have to pay to no more than 1 million won ($920)*, *irrespective of the costs patients incur while receiving treatment*. *Currently Park only suggests that the government will cover full costs for people receiving treatment for cancer*, *cardiovascular disorders*, *cerebrovascular disorders*, *and intractable diseases*. *The UPP candidate has much in common with Moon regarding health policies*, *recommending that hospitals be prohibited from taking more than 1 million won from individual patients each year*, *while also advocating that free medical treatment be made available for children aged younger than seven years old*. [10]

*“It is the duty of the state to protect the people’s health*,*” Moon said*. *“I will become a president who protects the people’s right to health and the patient’s right to life*.*” Moon also promised to ensure patients don’t pay more than 1 million won (US$923) per year in medical fees by reviewing the national health insurance system*. [11]

On the other hand, while values are crucial, a key goal of a political party is generally to gain power—in a presidential system, by occupying more seats in the legislature and/or winning the presidency in a general vote. According to election theories such as “issue ownership,” “issue framing,” and “issue voting,” if a political party preempts issues that people find important in an election campaign, this will help that party achieve victory [12, 13]. In the 2012 Korean election campaign, the opposition party was perceived as better able to handle two main issues, “economic democratization” and “social welfare expansion.” However, the election was won by the conservative candidate, Park, contrary to the belief in the opposition party that victory would come via effective issue ownership and issue framing. Thus, the effect of these practices and the influence of issue voters and candidates’ comments on or pledges to address certain issues, in particular health issues, remains unclear.

Many studies have delved into the relation between healthcare policies and voting behavior in general [14–16], but little is known about the influence of politicians’ welfare expansion pledges and handling of social welfare issues on the 2012 election outcome in Korea in particular. Our study had two main, interrelated purposes: to identify the factors influencing voters’ attitudes towards increasing public expenditure on health, and to identify whether and how the issue of healthcare influenced voting choice in the 2012 Korean presidential election.

## Methods

### Data source

The study used data from a survey conducted by the Institute of Korean Politics (IKP) at Seoul National University immediately following the 2012 presidential election. The IKP provides the data from this survey to the public and researchers. More detailed information such as the survey design, questionnaires, and raw datasets is available at the IKP website (http://www.ikps.or.kr/board03/view.asp?page=1&Key=1&ref1=1). A nationwide sample of 1,200 people was chosen to represent the entire Korean voting public (aged 19 or over). This survey used a multi-stage stratified randomized sampling method in which, first, proportionate quota sampling was performed in order to accurately reflect the distribution of sex, age, and region. After allocating these quotas, randomized sampling was conducted to secure a participant sample. Last, face-to-face interviews were performed with the selected participants. The standard error of this survey was ± 2.8% at the 95% confidence level. Among the 1,200 survey participants, we excluded 136 people who did not vote in the presidential election and also another 142 people who did not finish all the survey questions; thus, ultimately, the data from 922 participants were analyzed (S1 and S2 Files).

### Variables

The dependent variable used in this study was the candidate whom the voters supported—the conservative Park Geun-hye (coded as “1”) or the liberal Moon Jae-in (coded as “0”). Park and Moon respectively received 51.55% and 48.02% of the votes in the election, which elected Park president. In all, six candidates ran for the presidency in the 2012 elections, but the other four candidates accounted for very few of the total votes, and thus are excluded from our analysis.

The independent variable was people’s attitudes toward increase in public expenditure on healthcare. The response items, placed on a five-point Likert-type scale, were “expenditure should be greatly increased,” “expenditure should be somewhat increased,” “expenditure should remain the same,” “expenditure should be somewhat reduced” and “expenditure should be greatly reduced.” To facilitate analysis as a binary variable, these attributes were grouped into two categories—“expenditure should be greatly increased” and “expenditure should be somewhat increased” were grouped as “support increased expenditure,” whereas “expenditure should remain the same,” “expenditure should be somewhat reduced,” and “expenditure should be greatly reduced’ were grouped as “oppose increased expenditure.”

The control variables were selected based on previous study on the impact of voters’ attitudes toward economic democratization on their voting behavior [17]. They include variables relating to *voter ideology*, *party identification*, *political knowledge*, *perceptions on economic inequality*, *perceptions on household economy*, and *sociodemographic* characteristics of voters. In South Korea, the conservative and liberal parties have overwhelming political influence, while that of the progressive party is very limited and its position vulnerable. Therefore, in order to reflect voters’ political attitude, we used “ideology” as well as “party identification.” Voters’ political ideology was measured on an 11-point scale, with higher values given to the most conservative voters (i.e., coded very progressive as “0,” neutral as “5,” very conservative as “11”). Party identification measured whether the voter identified him/herself more closely with the conservative party or the liberal party. Respondents who selected the party of the liberal candidate were coded as “1,” and those who selected the party of the conservative candidate were coded as “5.” For voters who did not identify with any particular party, another question was asked: “Although you may not identify yourself with any one party, is there one party that you are more likely to identify with?” If the response named the party of the liberal candidate, the respondent was coded as “2,” and “4” for the party of the conservative candidate. Respondents who still did not identify with either party in the second questions were coded as “3,” the median value.

Political knowledge measured how much factual knowledge the respondents had on South Korean politics. The questions asked them to name the prime minister and the Speaker of the National Assembly at the time of the presidential election and to give the amount of the government budget for year 2012, and measured their knowledge by the number of correct answers.

As for perceptions on economic inequality, these questions were: “Do you believe the gap in income has increased between high-income earners and low-income earners compared to 5 years ago?” and “Do you believe that conflict between high-income earners and low-income earners has increased compared to 5 years ago?” Similarly, perceptions on household economy were measured based on answers to question: “Do you believe your household economy has improved over the past 5 years?” These questions were again answered on a five-point scale.

Sociodemographic variables covered age, educational attainment, income level, employment status, marital status, and sex. Additionally, place of residence was included, because voters’ regional background was closely related to the election results.

### Ethics statement

This study was exempted from approval by the Seoul National University Boramae Medical Center Institutional Review Board (IRB No. 07–2016–16).

### Statistical analysis

We performed a univariate analysis using the χ^2^ test and t-test to identify any relationships between candidate choice and independent, control, and socioeconomic variables. Then, to identify the factors determining attitudes towards public spending on health as well as the factors influencing presidential candidate choice, we conducted a logistic regression analysis. We tested the possibility of multicollinearity among independent variables using the correlation matrix, variance inflation factor (>2.5), condition index (>30), and variance decomposition proportions (>0.5) [18–20]. SPSS (version 22.0 K for Windows; SPSS Inc., Chicago) was used to perform all the statistical analyses. All tests were two-sided, and a p-value <0.1 was considered statistically significant.

## Results

There were significant differences in which candidate respondents supported according to age, education, income level, employment status, marriage, residing regions, and other variables. Regarding the prospect of increasing public expenditure on health, 44.3% of respondents supported it while 55.7% were opposed the issue. However, there was no significant difference in choice of candidate according to attitude towards increasing public expenditure on health (that is, 44.9% of Park voters and 43.6% of Moon voters supported an increase) (Table 1).

**Table 1:** General Characteristics of Study Population According to the Candidate Voters Supported.

Variables		Total (N)	Supporting candidate (%)		p-value
			Park Geun-hye (conservative)	Moon Jae-in (liberal)	
**Total**		922	56.7	43.3	
**Gender**	**Male**	452	55.1	44.9	0.325
	**Female**	470	58.3	41.7	
**Age (years)**	**20–39**	315	42.5	57.5	**<0.001**
	**40–59**	401	59.1	40.9	
	**60–**	206	73.8	26.2	
**Education**	**High school or lower**	160	68.1	31.9	**<0.001**
	**High school graduate**	296	62.8	37.2	
	**College or higher**	466	48.9	51.1	
**Income level**	**Low (<2 million KRW)**	151	66.9	33.1	**0.004**
	**Middle (2–<4 million KRW)**	401	57.9	42.1	
	**High (4 or over million KRW)**	370	51.4	48.6	
**Employment status**	**Employed**	670	58.5	41.5	**0.075**
	**Unemployed**	252	52.0	48.0	
**Marital status**	**Married**	699	59.9	40.1	**<0.001**
	**Others**	223	46.6	53.4	
**Residence**	**Seoul**	197	47.7	52.3	**<0.001**
	**Incheon, Gyeonggi**	209	63.2	36.8	
	**Daejeon, Chungbuk, Chungnam**	101	64.4	35.6	
	**Gwangju, Jeonbuk, Jeonnam**	108	13.0	87.0	
	**Daegu, Gyeongbuk**	110	90.0	10.0	
	**Busan, Ulsan, Gyeongnam**	164	58.5	41.5	
	**Gangwon, Jeju**	33	69.7	30.3	
**Attitude towards increasing public expenditure on health**	**Oppose**	513	56.1	43.9	0.688
	**Support**	409	57.5	42.5	
**Ideology (mean±SD)**		5.3±1.8	5.9±1.7	4.4±1.6	**<0.001**
**Party identification**	**Strong liberal party supporter**	183	3.8	96.2	**<0.001**
	**Weak liberal party supporter**	85	15.3	84.7	
	**Neutral**	250	47.6	52.4	
	**Weak conservative party supporter**	67	91.0	9.0	
	**Strong conservative party supporter**	337	95.8	4.2	
**Political knowledge (Number of correct answers)**	**0**	452	58.0	42.0	0.149
	**1**	257	57.2	42.8	
	**2**	158	57.6	42.4	
	**3**	55	41.8	58.2	
**Perceptions on economic gap between high- and low-income earners**	**Greatly increased**	294	55.1	44.9	0.682
	**Somewhat increased**	505	58.0	42.0	
	**Not increased**	123	55.3	44.7	
**Perceptions on conflict between high- and low-income earners**	**Greatly increased**	211	55.0	45.0	**0.056**
	**Somewhat increased**	514	59.9	40.1	
	**Not increased**	197	50.3	49.7	
**Perceptions on household economy changes in last 5 years**	**Improved**	84	59.5	40.5	0.529
	**Not changed**	457	58.0	42.0	
	**Somewhat worsened**	293	53.2	46.8	
	**Greatly worsened**	88	59.1	40.9	

If the difference is statistically significant at the p<0.1, p-values are marked in bold; p-values for all variables but ideology were calculated by χ^2^ test, while p-value for ideology was calculated by t-test.

To identify the determinants of respondents’ attitudes toward public health expenditure, we performed a logistic regression analysis. In Model 1, the middle-aged group (aged 40–59) showed negative attitudes toward increase in public health expenditure (OR = 0.73, 90% CI 0.54–0.99), while residents of Incheon/Gyeonggi (OR: 2.03) and Gangwon/Jeju (OR: 4.11) showed positive attitudes; all other age groups and regions were neutral. In the second model, which included the political spectrum variables, the results show that the more ideologically conservative a respondent, the greater their chance of opposing increased public expenditure on health (OR = 0.92, 90% CI 0.86–0.99). The age effect disappeared in Model 2, and politically neutral respondents were 1.66 times more likely to support the issue. In the third model, economic perceptions and other related variables were added. Neutrals (OR = 1.76, 90% CI 1.22–2.54) and strong conservative party supporters (OR = 1.53, 90% CI 1.05–2.25) were more likely to support public health expenditure increase compared to strong liberal party supporters. Also, respondents who believe that the economic gap was widening were 1.91 times more likely to support increase in public health expenditures (Table 2).

**Table 2:** Factors Affecting Attitudes towards Increasing Public Expenditure on Health.

Variables		Model 1		Model 2		Model 3	
		OR	90% CI	OR	90% CI	OR	90% CI
**Gender**	**Male**	1.00		1.00		1.00	
	**Female**	0.99	0.78–1.26	1.02	0.79–1.31	1.01	0.78–1.30
**Age (years)**	**20–39**	1.00		1.00		1.00	
	**40–59**	**0.73**	**0.54–0.99**	0.78	0.57–1.06	0.73	0.53–1.00
	**60–**	1.05	0.69–1.61	1.19	0.77–1.86	1.15	0.73–1.79
**Education**	**High school or lower**	1.00		1.00		1.00	
	**High school graduate**	1.08	0.72–1.61	1.07	0.71–1.59	1.06	0.71–1.59
	**College or higher**	1.21	0.78–1.88	1.15	0.74–1.80	1.13	0.72–1.78
**Income level**	**Low (<2 million KRW)**	1.00		1.00		1.00	
	**Middle (2–<4 million KRW)**	0.94	0.65–1.36	0.92	0.63–1.34	0.91	0.62–1.33
	**High (4 or over million KRW)**	0.72	0.48–1.09	0.73	0.48–1.11	0.76	0.50–1.16
**Employment status**	**Unemployed**	1.00		1.00		1.00	
	**Employed**	1.04	0.79–1.37	1.03	0.78–1.37	0.98	0.74–1.30
**Marital status**	**Married**	1.00		1.00		1.00	
	**Others**	0.98	0.73–1.33	0.97	0.71–1.32	0.95	0.70–1.30
**Residence**	**Seoul**	1.00		1.00		1.00	
	**Incheon, Gyeonggi**	**2.03**	**1.44–2.86**	**2.10**	**1.48–2.98**	**1.98**	**1.38–2.85**
	**Daejeon, Chungbuk, Chungnam**	0.89	0.58–1.36	0.81	0.52–1.24	0.87	0.56–1.35
	**Gwangju, Jeonbuk, Jeonnam**	1.27	0.84–1.92	1.38	0.89–2.16	**1.63**	**1.03–2.58**
	**Daegu, Gyeongbuk**	0.78	0.51–1.20	0.80	0.52–1.24	0.78	0.50–1.21
	**Busan, Ulsan, Gyeongnam**	1.23	0.86–1.77	1.21	0.84–1.75	1.17	0.81–1.70
	**Gangwon, Jeju**	**4.11**	**2.04–8.27**	**3.99**	**1.97–8.06**	**3.68**	**1.80–7.53**
**Ideology**				**0.92**	**0.86–0.99**	**0.92**	**0.86–0.99**
**Party identification**	**Strong liberal party supporter**			1.00		1.00	
	**Weak liberal party supporter**			1.30	0.83–2.06	1.24	0.78–1.97
	**Neutral**			**1.66**	**1.16–2.38**	**1.76**	**1.22–2.54**
	**Weak conservative party supporter**			1.41	0.84–2.37	1.51	0.89–2.56
	**Strong conservative party supporter**			1.35	0.93–1.96	**1.53**	**1.05–2.25**
**Political knowledge (Number of correct answers)**	**0**					1.00	
	**1**			0.95	0.72–1.25	0.97	0.73–1.28
	**2**			1.08	0.76–1.53	1.05	0.74–1.49
	**3**			1.15	0.69–1.93	1.13	0.67–1.91
**Perceptions on economic gap between high- and low-income earners**	**Not increased**					1.00	
	**Somewhat increased**					1.35	0.88–2.07
	**Greatly increased**					**1.91**	**1.16–3.15**
**Perceptions on conflict between high- and low-income earners**	**Not increased**					1.00	
	**Somewhat increased**					0.90	0.64–1.27
	**Greatly increased**					0.95	0.60–1.52
**Perceptions on household economy changes in last 5 years**	**Improved**					1.00	
	**Not changed**					1.18	0.77–1.81
	**Somewhat worsened**					1.41	0.90–2.21
	**Greatly worsened**					1.28	0.73–2.22
**Constant**		0.73		0.78		0.49	
**Nagelkerke R^2^**		0.060		0.072		0.088	

OR: odds ratio; CI: confidence interval. If the difference is statistically significant at p<0.1, OR and 90% CI are marked in bold.

Table 3 showed the factors affecting vote choice. In model 1, the old age group, employed people, and all regions excepting Gwangju/Jeonbuk/Jeonnam and Busan/Ulsan/Gyeongnam were more likely to vote for the conservative candidate. In model 2, which included variables for political attributes, participants who were more conservative ideologically and who identified as conservative party supporters were more likely to support the conservative candidate. Perceptions of a growing economic gap between high- and low-income earners had a negative effect on choice of the conservative candidate, while perceptions of conflict between high- and low-income earners had a positive effect on voting for the conservative candidate. Level of political knowledge and perceptions on household economy had no impact on candidate selection. The third model added variables on attitudes toward public expenditure on health: those who support increased public spending on health were more likely to support the conservative candidate, but the difference was not statistically significant (OR = 1.31, 90% CI 0.89–1.93) (Table 3). In the collinearity test, the maximum absolute value of the correlation coefficient was 0.68, for both perceptions of economic gap and perceptions of conflict. The maximal variance inflation factor was 1.91, and the maximal condition index was 44.11. When the condition index was 19.07, the corresponding variance decomposition proportions for perceptions of the economic gap and perceptions of conflict were 0.82 and 0.85, respectively. These results are unlikely to have been affected seriously by the mild collinearity.

**Table 3:** Factors Affecting Vote for the Conservative Presidential Candidate.

Variables		Model 1		Model 2		Model 3	
		OR	90% CI	OR	90% CI	OR	90% CI
**Gender**	**Male**	1.00		1.00		1.00	
	**Female**	1.22	0.93–1.60	1.31	0.86–1.98	1.30	0.86–1.97
**Age (years)**	**20–39**	1.00		1.00		1.00	
	**40–59**	**1.78**	**1.29–2.46**	1.58	0.97–2.59	1.63	0.99–2.67
	**60–**	**4.63**	**2.78–7.72**	**6.42**	**2.78–14.80**	**6.36**	**2.75–14.72**
**Education**	**High school or lower**	1.00		1.00		1.00	
	**High school graduate**	1.10	0.68–1.78	**2.68**	**1.22–5.85**	**2.62**	**1.19–5.76**
	**College or higher**	0.66	0.40–1.10	1.68	0.76–3.76	1.65	0.73–3.69
**Income level**	**Low (<2 million KRW)**	1.00		1.00		1.00	
	**Middle (2–<4 million KRW)**	0.92	0.59–1.46	1.18	0.58–2.37	1.21	0.60–2.44
	**High (4 or over million KRW)**	1.09	0.67–1.78	1.93	0.91–4.08	2.04	0.96–4.32
**Employment status**	**Unemployed**	1.00		1.00		1.00	
	**Employed**	**1.43**	**1.04–1.97**	**1.65**	**1.05–2.61**	**1.67**	**1.06–2.63**
**Marital status**	**Married**	1.00		1.00		1.00	
	**Others**	0.78	0.56–1.09	1.13	0.69–1.85	1.12	0.69–1.84
**Residence**	**Seoul**	1.00		1.00		1.00	
	**Incheon, Gyeonggi**	**2.03**	**1.41–2.91**	**3.61**	**1.99–6.53**	**3.48**	**1.91–6.32**
	**Daejeon, Chungbuk, Chungnam**	**2.01**	**1.29–3.15**	**5.04**	**2.54–10.03**	**5.08**	**2.55–10.11**
	**Gwangju, Jeonbuk, Jeonnam**	**0.11**	**0.06–0.19**	0.77	0.33–1.80	0.77	0.33–1.79
	**Daegu, Gyeongbuk**	**11.93**	**6.49–21.93**	**9.29**	**4.12–20.94**	**9.47**	**4.21–21.27**
	**Busan, Ulsan, Gyeongnam**	1.41	0.97–2.06	1.30	0.72–2.35	1.31	0.72–2.36
	**Gangwon, Jeju**	**2.21**	**1.08–4.52**	**5.01**	**1.72–14.58**	**4.71**	**1.60–13.87**
**Ideology**				**1.45**	**1.28–1.64**	**1.45**	**1.28–1.65**
**Party identification**	**Strong liberal party supporter**			1.00		1.00	
	**Weak liberal party supporter**			**7.04**	**2.92–16.95**	**7.05**	**2.92–16.99**
	**Neutral**			**29.23**	**13.82–61.83**	**29.27**	**13.82–62.01**
	**Weak conservative party supporter**			**289.37**	**99.24–843.74**	**294.52**	**100.72–861.25**
	**Strong conservative party supporter**			**642.34**	**263.43–1566.28**	**651.62**	**266.37–1594.04**
**Political knowledge (Number of correct answers)**	**0**			1.00		1.00	
	**1**			0.73	0.46–1.18	0.72	0.45–1.16
	**2**			0.69	0.38–1.26	0.67	0.36–1.22
	**3**			0.63	0.22–1.78	0.61	0.21–1.74
**Perceptions on economic gap between high- and low-income earners**	**Not increased**			1.00		1.00	
	**Somewhat increased**			**0.43**	**0.21–0.87**	**0.42**	**0.21–0.86**
	**Greatly increased**			0.46	0.20–1.03	**0.44**	**0.20–1.00**
**Perceptions on conflict between high- and low-income earners**	**Not increased**			1.00		1.00	
	**Somewhat increased**			**2.59**	**1.47–4.56**	**2.60**	**1.47–4.58**
	**Greatly increased**			**2.28**	**1.06–4.90**	**2.27**	**1.06–4.88**
**Perceptions on household economy changes in last 5 years**	**Improved**			1.00		1.00	
	**Not changed**			0.81	0.37–1.78	0.82	0.37–1.80
	**Somewhat worsened**			1.32	0.58–3.01	1.35	0.59–3.09
	**Greatly worsened**			0.64	0.25–1.64	0.65	0.25–1.68
**Attitude towards increasing public expenditure on health**	**Oppose**					1.00	
	**Support**					1.31	0.89–1.93
**Constant**		0.48		0.00		0.00	
**Nagelkerke R^2^**		0.321		0.743		0.744	

OR: odds ratio; CI: confidence interval. If the difference is statistically significant at p<0.1, OR and 90% CI are marked in bold.

We performed logical regression analysis to identify factors influencing candidate choice among political independents, that is, those who indicated they were party neutral. The results were similar to those from analysis of all respondents. Although attitude towards increasing public expenditure on health had a positive effect on choice of the conservative candidate (OR: 1.15), it was not statistically significant (Table 4).

**Table 4:** Factors Affecting Vote for the Conservative Presidential Candidate among Party Neutrals.

Variables		Model 1		Model 2		Model 3	
		OR	90% CI	OR	90% CI	OR	90% CI
**Gender**	**Male**	1.00		1.00		1.00	
	**Female**	1.16	0.71–1.90	1.05	0.62–1.79	1.04	0.61–1.78
**Age (years)**	**20–39**	1.00		1.00		1.00	
	**40–59**	**1.92**	**1.08–3.41**	**1.99**	**1.08–3.68**	**2.02**	**1.09–3.74**
	**60–**	**33.61**	**5.47–206.36**	**32.07**	**4.97–207.08**	**31.85**	**4.91–206.65**
**Education**	**High school or lower**	1.00		1.00		1.00	
	**High school graduate**	**20.88**	**3.26–133.67**	**27.45**	**3.93–191.79**	**26.35**	**3.75–185.31**
	**College or higher**	**11.59**	**1.78–75.64**	**16.33**	**2.29–116.36**	**15.83**	**2.21–113.34**
**Income level**	**Low (<2 million KRW)**	1.00		1.00		1.00	
	**Middle (2–<4 million KRW)**	0.84	0.31–2.27	0.92	0.33–2.59	0.95	0.34–2.68
	**High (4 or over million KRW)**	1.12	0.40–3.14	1.08	0.37–3.19	1.13	0.38–3.39
**Employment status**	**Unemployed**	1.00		1.00		1.00	
	**Employed**	**1.98**	**1.14–3.44**	**2.00**	**1.12–3.56**	**2.02**	**1.13–3.61**
**Marital status**	**Married**	1.00		1.00		1.00	
	**Others**	1.05	0.59–1.86	1.19	0.65–2.18	1.18	0.64–2.17
**Residence**	**Seoul**	1.00		1.00		1.00	
	**Incheon, Gyeonggi**	**2.41**	**1.21–4.80**	**3.23**	**1.52–6.87**	**3.22**	**1.52–6.85**
	**Daejeon, Chungbuk, Chungnam**	**3.28**	**1.56–6.93**	**4.14**	**1.81–9.48**	**4.20**	**1.83–9.62**
	**Gwangju, Jeonbuk, Jeonnam**	0.76	0.25–2.32	1.03	0.30–3.61	1.02	0.29–3.56
	**Daegu, Gyeongbuk**	**6.15**	**2.40–15.80**	**6.84**	**2.54–18.46**	**7.02**	**2.59–18.99**
	**Busan, Ulsan, Gyeongnam**	1.27	0.59–2.71	1.52	0.67–3.44	1.53	0.68–3.45
	**Gangwon, Jeju**	2.83	0.81–9.87	**4.30**	**1.13–16.32**	**4.17**	**1.09–16.00**
**Ideology**				**1.28**	**1.08–1.51**	**1.28**	**1.09–1.51**
**Political knowledge (Number of correct answers)**	**0**			1.00		1.00	
	**1**			0.94	0.49–1.81	0.93	0.48–1.79
	**2**			0.97	0.44–2.12	0.95	0.43–2.09
	**3**			0.76	0.15–3.91	0.77	0.15–3.90
**Perceptions on economic gap between high- and low-income earners**	**Not increased**			1.00		1.00	
	**Somewhat increased**			0.76	0.33–1.74	0.75	0.33–1.72
	**Greatly increased**			0.64	0.24–1.73	0.62	0.23–1.70
**Perceptions on conflict between high- and low-income earners**	**Not increased**			1.00		1.00	
	**Somewhat increased**			1.97	0.96–4.02	1.98	0.97–4.05
	**Greatly increased**			1.24	0.46–3.36	1.24	0.46–3.36
**Perceptions on household economy changes in last 5 years**	**Improved**			1.00		1.00	
	**Not changed**			0.86	0.29–2.51	0.86	0.29–2.51
	**Somewhat worsened**			0.96	0.31–3.01	0.98	0.31–3.07
	**Greatly worsened**			0.61	0.17–2.15	0.62	0.18–2.18
**Attitude towards increasing public expenditure on health**	**Oppose**					1.00	
	**Support**					1.15	0.69–1.90
**Constant**		0.01		0.00		0.00	
**Nagelkerke R^2^**		0.244		0.295		0.295	

OR: odds ratio; CI: confidence interval. If the difference is statistically significant at p<0.1, OR and 90% CI are marked in bold.

## Discussion

A presidential election is a political arena that reflects the prevailing zeitgeist. The solutions to the major election issues that are raised by a candidate and his or her party during elections will be the foundation of future national policy if they are elected. In this sense, the election itself is an important political action, setting the policy direction of the country. Economic justice and welfare issues have not traditionally been major issues in presidential elections in Korea. However, this changed in the 2012 election, when economic democratization and social welfare expansion became hot issues [1–3].

Our first research question was how many constituents supported increasing public health expenditure and what factors positively and negatively affected their attitudes toward doing so. About 44.3% of respondents supported an increase. A more interesting finding is there was no significant difference in attitudes by which candidates the respondent voted for. Many different factors no doubt influence attitudes on welfare services, including sociodemographic factors such as sex, age, education level, occupation, social class, and income [21–26], and they cannot be reduced to one’s general political leadings; nevertheless, this lack of a gap is an extraordinary phenomenon, as traditionally, conservatives are likely to have negative attitudes toward social welfare services. In order to investigate what factors affecting attitudes on expanding public health expenditure, we constructed three models and analyzed each of them using logistic regression. Respondents who believed that the economic gap in their society was widening were 1.91 times more likely to support an increase in public health expenditures than those who did not. Meanwhile, controlling for other factors, perception of increasing economic inequality also had a significant positive effect on attitudes toward increased public expenditure on health. Such result coincides with previous results from another study conducted in Korea [27]. However, a novel and interesting finding here is that neutrals (OR: 1.76) and strong conservative party supporters (OR: 1.53) were more likely to show positive attitudes toward expanding public health spending compared to strong liberal party supporters (Table 2). In general in Western countries, low-income, working-class, less educated, and women voters have a tendency to support welfare expansion [23, 25, 28, 29]. In contrast, the common perception in Korea has been that welfare attitudes are determined more by political stance than by social class [30, 31]. That is, conservatives in Korea have usually taken negative positions on social welfare expansion regardless of their own socioeconomic position. However, our results showed that conservative constituents had positive attitudes to the expansion of healthcare. This finding might mean that the majority of Koreans have begun to reach a consensus on the issue of social welfare expansion, that is, to agree that it is needed to create a better social safety net; the global economic crisis of recent years may certainly have helped foster this consensus. However, it is hard to interpret the results with great confidence using our current dataset only. Therefore, further research is needed.

The second question was on the relationship between attitudes toward welfare expansion and voting choice, and specifically whether a positive attitude toward welfare expansion could have a negative effect on choice of a conservative presidential candidate. In theory, debate on the issue might have a positive effect on public expenditure on health. Then, constituents who had a positive attitude on an increase would be more likely to vote for the liberal party’s candidate, whereas there would be a negative effect on choosing the conservative candidate if constituents who identified their political position as “conservative” or “neutral or independent” had a positive position toward increased spending. However, these effects did not occur in our data. Instead, respondents supporting the increased public spending on health were slightly more likely to support the conservative candidate, although this difference was not statistically significant (OR = 1.31) (Table 3). For neutral or independent respondents in particular, there was again a non-significant positive effect (OR: 1.15) on choosing the conservative candidate (Table 4). In sum, our findings show that the issue had no meaningful influence on voter’s choice of presidential candidate.

How can we interpret our results? One possible interpretation is that constituents voted along partisan lines regardless of their attitude on welfare issues. According to previous research, ideology and party identification are major factors influencing candidate selection [11, 13, 32]. In particular, in Korea, partisanship is a stronger predictor of constituents’ voting behavior than the constituents’ socioeconomic position [30, 31]. Therefore, among voters supporting the conservative party, even if their perception of social welfare expansion has become more positive, it remains difficult for them to vote for the opposition party’s liberal candidate.

A second possible explanation is the success of the “left click strategy” in the conservative party. Economic democratization and social welfare issues would seem more naturally to fall under the purview of the liberal party [12] from the perspective of “issue ownership” and “issue framing.” However, the conservative party may have succeeded in sharing in the potential benefit to be gained from engaging with these issues by moving their policies toward the left and to some degree embracing economic democratization and social welfare expansion. The only differences in the two parties’ positions in the 2012 election were the degree of change and the pace of policy implementation—the “customized welfare” of the conservative versus the “universal welfare” of the liberal [1]. The main issues were not “owned” by the liberal party anymore. Such a change in strategy might have a considerable effect, if it opened the possibility of politically independent or neutral constituents motivated by specific issues to vote for the conservative candidate instead of the liberal candidate, while still potentially voting for the liberal candidate if the conservative party refused to address the main issues. Also, for the supporters of the conservative, this frame change would provide a more comfortable environment, allowing them to vote for the conservative candidate even though they were in favor of expanding social welfare.

Some limitations do exist to our study, however. First, the preconditions of an issue voting are basically that voters have sufficient knowledge of an issue, that candidates have a clear difference in their stances towards the issue, and that voters are aware of this difference [32]. The need for improving the benefit coverage of NHI was certainly a widely recognized issue among voters, and they were sufficiently informed of the pledges made by the two leading candidates through debates on TV, media coverage, and the marketing activities of the parties. The candidates had different approaches to improving the benefit coverage—“cost-oriented” vs. “specific diseases.” However, there was no data available on the level of awareness among voters of how the two pledges differed from another. This topic was not covered in the data used in this study, and, thus, was excluded from the variables for analysis. It is essential to include level of voter awareness of pledges as a variable for analysis in future studies. The survey used in our study did not specifically investigate attitudes towards pledges to improve the benefit coverage of the NHI, but during the election campaign period, debates did occur around the pledges of the two candidates on this issue and the appropriate the size of public expenditure. Voters perceived the pledge from the liberal candidate to be in support of expanding public expenditure and the pledge from the conservative candidate to be passive towards increased government spending. Hence, using attitude towards increased public spending on health as a proxy variable to describe attitudes towards the pledges by the two candidates does not seem to present much difficulty. In the US, in contrast, political campaign messages often have a large impact on voters due to extensive media exposure. However, we did not use a media exposure variable as a control because there is no relevant question in the survey questionnaire. We admit that this might be a weak point of this study. Therefore, in future work, we should include a media exposure factor [33].

To sum up, the main findings of this study are as follows: political ideology and perceptions of economic inequality had significant influences on attitudes towards increased public spending on health. The factors that influenced choice of candidates were age, sex, political ideology, and party identification, but attitudes toward public spending on health, which were the subject of our main interest in this study, did not have a statistically significant role. We observed similar results in our analysis of political independents alone, suggesting that they are not specific to supporters of particular parties.

During the 2012 Korean presidential election, the camps for each candidate, relevant experts, and the media all expected that pledges from the candidates on public healthcare would have direct impact on voting behavior. Healthcare pledges were at the center of policy debate among the leading candidates throughout the election campaign period. But, the findings of our study suggest that issue voting in healthcare did not take place to a meaningful degree during the 2012 election. Previous literature has shown that both policy preference and party identification are determinants of candidate selection, but in the case of Korea, where welfare attitudes are inconsistent and cross-cutting in terms of class and where one’s position on the political-ideological spectrum has a strong influence, policy preference appears to not play a significant role in candidate choice among voters.

## Supporting Information

S1 File Data for analysis. (XLSX)

S2 File Variables and codes. (XLSX)
